# Influence of Preoperative Cognitive Function, Self-Efficacy, and Postoperative Psychological Counselling on Anxiety and Depression Levels in Patients Undergoing Botulinum Toxin Injections

**DOI:** 10.62641/aep.v54i2.2215

**Published:** 2026-04-15

**Authors:** Xiaoli Yu, Yumin Zhang, Liangyu Zhang, Tiejun Chen, Bin Yao

**Affiliations:** ^1^Department of Medical Cosmetology, Huzhou Maternity and Child Health Care Hospital, 313000 Huzhou, Zhejiang, China; ^2^Department of Dermatology, Huzhou Maternity and Child Health Care Hospital, 313000 Huzhou , Zhejiang, China

**Keywords:** cognitive function, self-efficacy, psychological counselling, botulinum toxin, injection

## Abstract

**Background:**

To investigate the prognostic influence of preoperative cognitive function, self-efficacy, and postoperative psychological counselling on treatment response following botulinum toxin injections in patients with anxiety and depressive disorders, and to identify key predictors of treatment response.

**Methods:**

A retrospective study was conducted on 176 patients who received botulinum toxin injections at Huzhou Maternity and Child Health Care Hospital between May and December 2025. Based on the treatment response of anxiety and depressive symptoms assessed eight weeks post-injection, participants were categorised into a responder group (*n* = 108) and a non-responder group (*n* = 68). Data collect included demographic characteristics, botulinum toxin injection details, psychological counselling records, pre-injection assessment of cognitive function and self-efficacy. Pearson correlation analysis was used to assess the relationship between preoperative cognitive levels and self-efficacy, and the effectiveness of postoperative psychological counselling on treatment outcomes of anxiety and depression. Multivariate logistic regression analysis was employed to identify factors influencing treatment response to anxiety and depression, and receiver operating characteristic (ROC) curves were used to evaluate the predictive performance of these factors.

**Results:**

Both study populations exhibited negative correlations between preoperative Pittsburgh Sleep Quality Index (PSQI) scores and Hamilton Anxiety Rating Scale (HAMA) reduction rates, as well as Hamilton Depression Rating Scale (HAMD) reduction rates. Conversely, preoperative Montreal Cognitive Assessment (MoCA) scores, preoperative self-efficacy, and duration per session showed positive correlations with HAMA reduction rates and HAMD reduction rates (all *p* < 0.05). Multivariate logistic regression analysis revealed that counselling frequency (OR = 3.808, β = 1.337), duration per session (OR = 1.092, β = 0.088), preoperative PSQI score (OR = 0.820, *β *= −0.198), MoCA (OR = 1.312, β = 0.272), and General Self-Efficacy Scale (GSES) score (OR = 1.175, β = 0.161) were identified as factors influencing treatment response of anxiety and depression following botulinum toxin injection (*p* < 0.05). ROC curve analysis indicated that the aforementioned variables possessed predictive value for treatment response. The combined predictive model yielded an area under the curve was 0.866 (95% confidence interval, ranging from 0.810 to 0.921).

**Conclusions:**

Preoperative cognitive function, self-efficacy and the duration per session were correlated with treatment response rates for anxiety and depression. Injection sites, counselling sessions, the duration per session, and preoperative PSQI, MoCA and GSES scores were identified as independent factors influencing treatment response following botulinum toxin injection.

## Introduction

With rapid advances in medical aesthetics, botulinum toxin injections have become one of the mainstreams minimally invasive treatments for improving facial dynamic wrinkles and contouring [1]. However, clinical practice reveals that among individuals receiving botulinum toxin injections, the prevalence of comorbid anxiety and depressive disorders reach as high as 33.8%. This high prevalence may not only diminish patient satisfaction with treatment outcomes but could also compromise treatment adherence [2, 3]. Consequently, investigating factors that influence the treatment response of anxiety and depressive symptoms following botulinum toxin injections holds remarkable importance for optimising clinical intervention strategies.

Currently, most existing research has focused on the impact of physiological factors—such as injection sites and dosage—on aesthetic outcomes, whereas the role of psychosocial factors remains relatively underexplored [4, 5]. Cognitive function shapes an individual’s cognitive assessment of treatment; intact cognitive function can reduce cognitive biases, such as unrealistic expectations [6]. Self-efficacy, defined as an individual’s belief in their own ability to cope, can regulate emotions through dual pathways: fostering positive coping strategies and enhancing self-identity [7]. Postoperative psychological counselling, defined as a targeted intervention for improving emotional well-being, can enhance patients’ self-management capabilities by providing avenues for emotional expression, correcting cognitive biases, and imparting emotion regulation techniques [8]. However, the mechanisms through which these three factors influence the treatment response for depression and anxiety following botulinum toxin injections, as well as their relationships, remain to be systematically validated.

To address this gap, this study retrospectively collected clinical data from patients receiving botulinum toxin injections who had comorbid anxiety or depression disorders. With treatment response at eight weeks post-procedure as the primary outcome, we investigated the correlation between preoperative cognitive function, self-efficacy, and postoperative psychological counselling and the treatment response of anxiety and depression. The study aimed to identify key influencing factors and thereby inform strategies to further optimise treatment outcomes for patients.

## Materials and Methods

### Research Subjects

A retrospective review was conducted of the clinical records for 176 patients who underwent botulinum toxin injections at Huzhou Maternity and Child Health Care Hospital between May and December 2025. With treatment response of anxiety and depressive symptoms as the primary outcome, the pre-trial effect size for the reference core variable (self-efficacy) was OR = 0.85. Setting the test level α = 0.05 (two-tailed) and test power 1–β = 0.80, the sample size calculations indicated a minimum of 152 patients was required. A total of 176 eligible patients were retrospectively identified, meeting the statistical power requirements. The inclusion criteria were as follows: (1) aged 18–60 years, gender unrestricted; (2) diagnosed with comorbid anxiety or depressive disorder by a psychiatrist or neurologist, with Hamilton Anxiety Rating Scale (HAMA) ≥14 points [9] and Hamilton Depression Rating Scale (HAMD) ≥8 points [10]; (3) complete clinical records; (4) voluntary acceptance of botulinum toxin injection for cosmetic treatment with no contraindications; and (5) for patient already receiving psychiatric medications prior to the procedure, the medication regimen must have been stable for ≥4 weeks, and the patient must have agreed to maintain the original regimen unchanged during the study period. For patients not taking psychiatric medications, the subject must have agreed not to initiate any new medications during the study period. The exclusion criteria were as follows (1) history of severe mental disorders such as schizophrenia or bipolar disorder; (2) contraindications to botulinum toxin injections or a history of allergy; (3) receipt of intensive intervention targeting the disorder during follow-up; (4) concurrent severe organic diseases (cardiac, hepatic, renal) or neurological pathology impairing symptom assessment; (5) incomplete clinical records; and (6) loss to follow-up. This study adhered to the Declaration of Helsinki [11] and was approved by the Ethics Committee of Huzhou Maternity and Child Health Care Hospital (ethics approval number: 2026-J-003). All participating patients were fully informed and provided written informed consent.

### Grouping Method

Patients were categorised into two groups based on treatment response of depressive and anxiety symptoms at eight weeks post-injection [12]. Responder group: Patient who achieved both a HAMA score reduction rate ≥50% and a HAMD score reduction rate ≥50% at the eight-week follow-up post injection were included in this group. The reduction rate was calculated as follows: (pre-operative baseline score−eight-week post-operative score) / pre-operative baseline score × 100%. A total of 108 patients met both criteria and were classified as responders. Non-responder group: patients who exhibited a HAMA score reduction rate <50%, and/or a HAMD score reduction rate was <50% at the eight-week follow-up post-injection were assigned to this group. Individuals failing to meet both responder criteria were classified as non-responders, comprising a total of 68 patients.

### Data Collection

The following data were extracted from the hospital electronic medical record systems, archived databases of psychological assessment scales, and postoperative follow-up management systems: (1) Demographic characteristics: gender, age, body mass index (BMI), educational attainment, occupation, marital status, and monthly household income. (2) Botulinum toxin injection-related characteristics: prior injection history (frequency) and injection sites. (3) Psychological counselling records: number of sessions and duration per session. (4) Anxiety and depressive disorders: Anxiety levels were assessed preoperatively and at eight weeks postoperatively using the HAMA scale, a 5-point scoring system (0–4) where higher scores indicate greater anxiety severity [13]. The Cronbach’s α for this scale is 0.832. Depression severity was assessed via the HAMD scale, also a 5-point scale (0–4), with higher scores indicating greater depression severity [14]. The Cronbach’s α for this scale is 0.810. (5) Sleep disorders: Pittsburgh Sleep Quality Index (PSQI) [15] was administered preoperatively. The total score ranges from 0 to 21, with higher scores indicating poorer sleep quality [16]. The Cronbach’s α for this scale is 0.810. (6) Cognitive impairment: Patients underwent Montreal Cognitive Assessment (MoCA) [17] preoperatively. The maximum score is 30 points, with higher scores indicating better cognitive function [18]. The Cronbach’s α for this scale is 0.730. (7) Self-efficacy: Patients completed General Self-Efficacy Scale (GSES) [19] preoperatively. The total score ranges from 10 to 40 points, with higher scores indicating greater self-efficacy [20]. The Cronbach’s α for this scale is 0.892.

### Statistical Analysis

All data analyses were conducted using SPSS 27.0 (IBM, Armonk, NY, USA). Normality of continuous variables was assessed using the Kolmogorov–Smirnov test. Variables with a normal distribution were expressed as mean ± standard deviation, while non-normality distributed variables were expressed as median (interquartile range). Intergroup comparisons were employed using independent samples t-tests or Mann–Whitney U tests. Categorical variables were presented as frequencies and percentages [*n* (%)], with intergroup comparisons performed using the chi–square test or Fisher’s exact probability test. Pearson correlation analysis was employed to evaluate the relationship between preoperative cognitive function, self-efficacy, and postoperative psychological counselling, and treatment response of anxiety and depressive symptoms following botulinum toxin injections. Multivariate logistic analysis was employed to investigate factors influencing the efficacy of botulinum toxin injections in treatment response of anxiety and depression. Study population served as the dependent variable, whilst preoperative PSQI, MoCA, and GSES scores, along with postoperative psychological counselling parameters, were included as independent variables. Additional factors were incorporated as confounding variables within univariate logistic regression analyses. Variables yielding *p* < 0.05 in univariate logistic regression were subsequently integrated to construct a Multivariate logistic regression model. This study employed a dual analytical strategy to evaluate the efficacy of treatment response of anxiety and depression. First, Pearson correlation analysis was conducted using the percentage reduction in HAMA and HAMD scores as continuous variables, preserving data integrity. Second, patients were grouped based on a reduction rate ≥50%, with group membership serving as the dependent variable in logistic regression analysis to identify independent predictors of clinical efficacy. These complementary approaches allow for both detailed continuous variable analysis and clinically meaningful categorical variable classification. Receiver operating characteristic (ROC) curves analysis was performed to assess the predictive efficacy of the identified factors. Figures and tables were generated using GraphPad Prism 10 (GraphPad Software, La Jolla, California, USA). A *p* < 0.05 was considered significant.

## Results

### Comparison of Two Sets of Baseline Data

No significant differences were observed between the responder and non-responder groups in terms of gender, age, BMI, occupation, marital status, previous history of botulinum toxin injection, injection site, or preoperative HAMA and HAMD scores (*p* > 0.05). However, significant differences were found between the two groups regarding educational level, monthly household income, number of counselling sessions, duration per session, and preoperative PSQI, MoCA and GSES scores (*p* < 0.05) (Table 1).

**Table 1.** **Comparison of baseline data**.

Indicator		Responder group (*n* = 108)	Non-responder group (*n* = 68)	χ^2^/*t*	*p*
Sex, *n* (%)				0.915	0.340
	Male	6 (5.56)	1 (1.47)		
	Female	102 (94.44)	67 (98.53)		
Age, year, mean ± SD		38.30 ± 8.13	37.77 ± 9.86	0.387	0.699
BMI, kg/m^2^, mean ± SD		24.20 ± 2.77	23.44 ± 2.43	1.857	0.065
Education level, *n* (%)				8.918	0.012
	Junior secondary and below	19 (17.59)	12 (17.65)		
	Senior secondary	33 (30.56)	35 (51.47)		
	Undergraduate degree and above	56 (51.85)	21 (30.88)		
Occupation, *n* (%)				4.221	0.239
	Government employee	20 (18.53)	21 (30.88)		
	Company employee	48 (44.44)	23 (33.82)		
	Freelancer	26 (24.07)	14 (20.59)		
	Unemployed	14 (12.96)	10 (14.71)		
Marital status, *n* (%)				0.285	0.593
	Married	98 (90.74)	60 (88.24)		
	Unmarried	10 (9.26)	8 (11.76)		
Monthly income (CNY), *n* (%)				8.314	0.040
	<3000	11 (10.18)	12 (17.65)		
	3000–5000	23 (21.30)	16 (23.53)		
	5000–10,000	40 (37.04)	31 (45.59)		
	>10,000	34 (31.48)	9 (13.23)		
Number of injections, mean ± SD		2.69 ± 1.13	2.82 ± 1.38	0.682	0.497
Injection site, *n* (%)				6.625	0.157
	Face	83 (76.85)	41 (60.30)		
	Neck	4 (3.70)	5 (7.35)		
	Masseter muscle	13 (12.04)	14 (20.59)		
	Underarms	3 (2.78)	5 (7.35)		
	Other areas	5 (4.63)	3 (4.41)		
Number of counselling sessions, *n* (%)				7.765	0.005
	<3 times	45 (41.67)	43 (63.24)		
	≥3 times	63 (58.33)	25 (36.76)		
Duration per session, min, mean ± SD		47.09 ± 11.90	37.15 ± 10.62	5.621	<0.001
HAMA, mean ± SD		28.43 ± 4.38	28.41 ± 4.40	0.029	0.977
HAMD, mean ± SD		15.26 ± 4.06	15.09 ± 3.42	0.287	0.775
PSQI, mean ± SD		9.69 ± 3.72	11.89 ± 3.30	3.987	<0.001
MoCA, mean ± SD		24.01 ± 2.24	23.06 ± 2.02	2.844	0.005
GSES, mean ± SD		28.38 ± 4.11	25.56 ± 3.90	4.520	<0.001

Note: SD, standard deviation; BMI, body mass index; HAMA, Hamilton Anxiety Rating Scale; HAMD, Hamilton Depression Rating Scale; PSQI, Pittsburgh Sleep Quality Index; MoCA, Montreal Cognitive Assessment; GSES, General Self-efficacy Scale. 1 USD = 6.88 CNY.

### The Correlation between Preoperative Cognitive Function, Sleep Disorders, and the Treatment Response of Anxiety and Depression

In both groups, significant correlations were observed between cognitive function, sleep disorders, self-efficacy, duration per session, and the reduction rates of HAMA and HAMD scores (all *p* < 0.05). Specifically, preoperative PSQI scores were negatively correlated with both the HAMA reduction rate and the HAMD reduction rate. Conversely, preoperative MoCA scores, preoperative GSES scores, and duration per session were positively correlated with both the HAMA reduction rate and the HAMD reduction rate (Table 2).

**Table 2.** **The correlation between preoperative cognitive function, sleep disorders, self-efficacy, and duration per session with the treatment response of anxiety and depression**.

Variables	HAMA reduction rate (*r*)	*p*	HAMD reduction rate (*r*)	*p*
PSQI	−0.224	0.003	−0.232	0.002
MoCA	0.185	0.026	0.188	0.031
GSES	0.267	<0.001	0.226	0.003
Duration per session	0.243	0.001	0.271	<0.001

Note: HAMA, Hamilton Anxiety Rating Scale; HAMD, Hamilton Depression Rating Scale; PSQI, Pittsburgh Sleep Quality Index; MoCA, Montreal Cognitive Assessment; GSES, General Self-efficacy Scale.

### Logistic Regression Analysis of the Efficacy of Botulinum Toxin Injections in Treatment Response of Anxiety and Depression

Preoperative PSQI, MoCA, and GSES scores, along with postoperative psychological counselling parameters, were included as independent variables. Additional factors presented in Tables 1 and 2 were incorporated as confounding variables. Multivariate logistic regression analysis revealed several factors as significant independent predictors of treatment response of anxiety and depression following botulinum toxin injection (*p* < 0.05). These included number of counselling sessions (OR = 3.808, β = 1.337), duration per session (OR = 1.092, β = 0.088), preoperative PSQI score (OR = 0.820, β = −0.198), preoperative MoCA score (OR = 1.312, β = 0.272), and preoperative GSES score (OR = 1.175, β = 0.161) (Table 3).

**Table 3.** **Logistic regression analysis of the efficacy of botulinum toxin injections in treatment response of anxiety and depression**.

		Univariate analysis				Multivariate analysis			
Variables		β	SE	*p*	OR (95% CI)	β	SE	*p*	OR (95% CI)
Sex									
	Male				1.000 (Reference)				
	Female	−1.371	1.092	0.209	0.254 (0.030–2.155)				
Age		0.007	0.018	0.698	1.007 (0.973–1.042)				
BMI		0.108	0.060	0.070	1.115 (0.991–1.253)				
Education level									
	Junior secondary				1.000 (Reference)				
	Senior secondary	−0.518	0.441	0.240	0.595 (0.251–1.414)				
	Undergraduate degree	0.521	0.449	0.245	1.684 (0.699–4.059)				
Occupation									
	Government employee				1.000 (Reference)				
	Company employee	0.784	0.402	0.051	2.191 (0.996–4.822)				
	Freelancer	0.668	0.456	0.143	1.950 (0.799–4.762)				
	Unemployed	0.385	0.519	0.458	1.470 (0.532–4.063)				
Marital status									
	Married				1.000 (Reference)				
	Unmarried	−0.162	0.493	0.743	0.851 (0.324–2.235)				
Monthly income (CNY)									
	<3000				1.000 (Reference)				
	3000–5000	0.450	0.529	0.395	1.568 (0.556–4.426)				
	5000–10,000	0.342	0.481	0.477	1.408 (0.548–3.614)				
	>10,000	1.416	0.561	0.062	4.121 (0.981–12.376)				
Number of injections		−0.090	0.125	0.470	0.914 (0.715–1.167)				
Injection site									
	Face				1.000 (Reference)				
	Neck	−0.928	0.697	0.183	0.395 (0.101–1.550)				
	Masseter muscle	−0.779	0.430	0.070	0.459 (0.198–1.065)				
	Underarms	−1.216	0.755	0.107	0.296 (0.068–1.301)				
	Other areas	−0.194	0.755	0.797	0.823 (0.188–3.615)				
Number of counselling									
	<3 times				1.000 (Reference)				1.000 (Reference)
	≥3 times	0.943	0.320	0.003	2.567 (1.970–4.807)	1.337	0.439	0.002	3.808 (1.612–8.996)
Duration per session		0.073	0.015	<0.001	1.076 (1.045–1.108)	0.088	0.020	<0.001	1.092 (1.051–1.134)
Pre-HAMA		0.001	0.035	0.983	1.001 (0.934–1.072)				
Pre-HAMD		0.012	0.041	0.773	1.012 (0.934–1.096)				
PSQI		−0.172	0.047	<0.001	0.842 (0.769–0.923)	−0.198	0.063	0.002	0.820 (0.725–0.927)
MoCA		0.208	0.076	0.006	1.231 (1.062–1.428)	0.272	0.102	0.008	1.312 (1.074–1.603)
GSES		0.176	0.043	<0.001	1.192 (1.096–1.297)	0.161	0.053	0.002	1.175 (1.060–1.303)

Note: OR, odds ratio; BMI, body mass index; Pre-HAMA, preoperative Hamilton anxiety rating scale; Pre-HAMD, preoperative Hamilton depression rating scale; PSQI, Pittsburgh Sleep Quality Index; MoCA, Montreal Cognitive Assessment; GSES, General Self-efficacy Scale. 1 USD = 6.88 CNY.

### ROC Curve Analysis of Factors Predicting Treatment Response of Anxiety and Depression Following Botulinum Toxin Injection

**Fig. 1.**
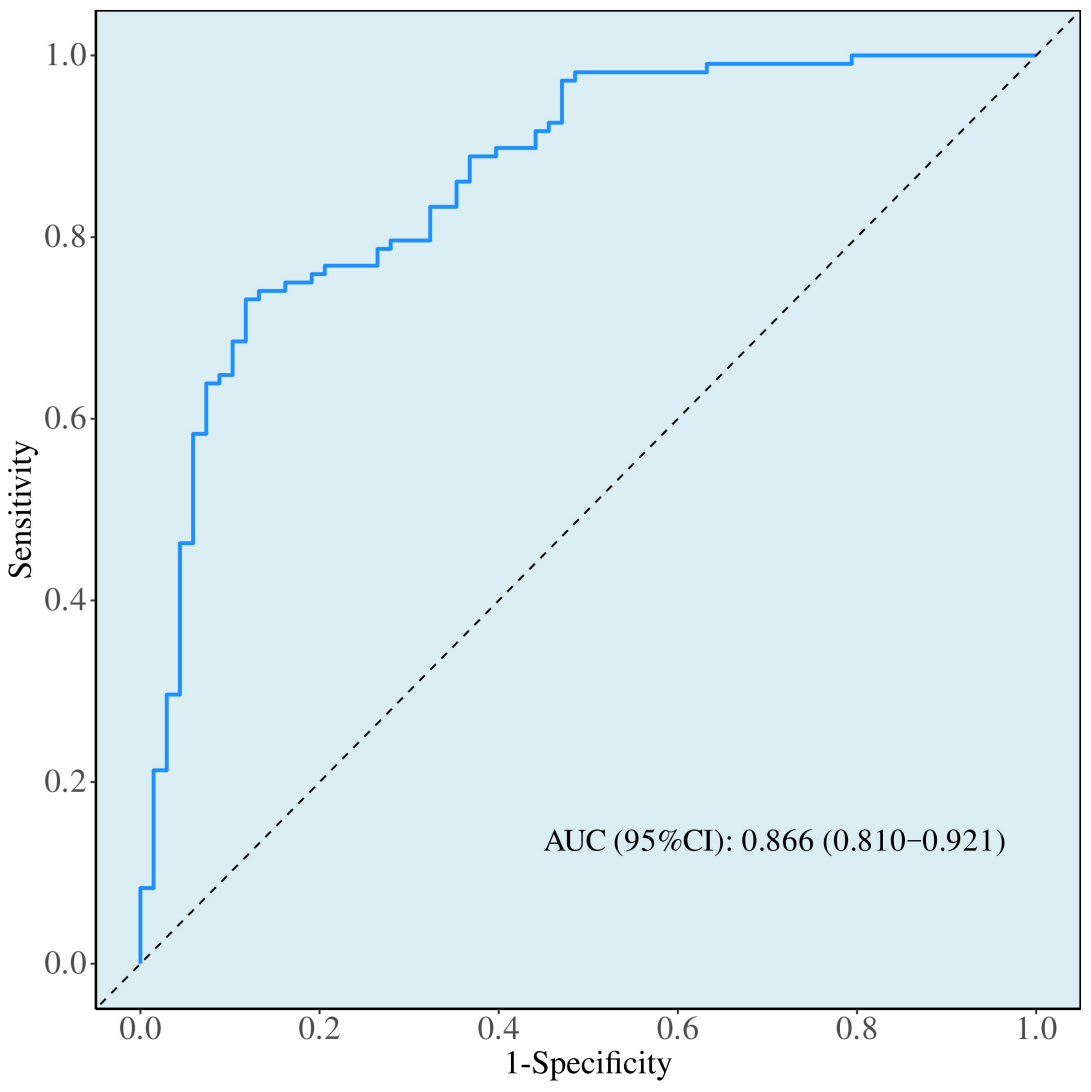
**ROC curve analysis for the efficacy of treatment response of depression and anxiety**. AUC, area under the curve; ROC, receiver operating characteristic.

ROC curve analysis demonstrated that the number of counselling sessions, duration per session, and preoperative PSQI, MoCA, and GSES scores possessed predictive value for treatment response of anxiety and depression following botulinum toxin injection. The combined predictive model yielded an area under the curve (AUC) of 0.866 > 0.7, with a 95% CI ranging from 0.810 to 0.921 (Fig. 1).

## Discussion

This study reviewed clinical data from patients receiving botulinum toxin injections who had comorbid anxiety or depressive disorders, and examined the influence of preoperative cognitive function, self-efficacy, and postoperative psychological counselling on the therapeutic response of anxiety and depression following the injections. The results indicated that preoperative cognitive function, self-efficacy, and the duration per session was correlated with the therapeutic response. Furthermore, injection site, number of counselling sessions, duration per session, and preoperative PSQI, MoCA and GSES scores were identified as independent factors influencing treatment response of anxiety and depression.

Preoperative cognitive function emerged as a key factor influencing patients’ treatment response for postoperative anxiety and depression. PSQI score were negatively correlated with both HAMA and HAMD reduction rate, whereas MoCA score demonstrates a positive correlation with both reduction rate. Patients exhibiting severe preoperative anxiety and depressive symptoms often harbour unrealistically high and demanding expectations regarding aesthetic treatment outcomes. Even when botulinum toxin injections achieve the desired cosmetic results, they may overlook positive changes due to persistent emotional distortions, potentially hindering the alleviation of anxiety and depressive symptoms [21, 22]. Sleep disturbances, as reflected by elevated PSQI scores, can disrupt the balance of neurotransmitters such as serotonin and dopamine, thereby reducing the sensitivity of the brain’s emotional regulation centres [23]. This impairment can hinder patients’ ability to disengage from negative emotions, while concurrent anxiety and depression further exacerbate sleep disorders, creating a vicious cycle [24]. Conversely, superior preoperative cognitive function was associated with more effective postoperative emotional relief, consistent with the findings of Yu *et al*. [25], who reported that cognitive function is positively correlated with emotional regulation capacity. Robust cognitive function enables patients to rationally assess the treatment cycle and efficacy limits of botulinum toxin injections, reducing unnecessary anxieties stemming from cognitive biases. It also facilitates better comprehension and acceptance of healthcare professionals’ healthcare guidance, which correlate emotional recovery.

Self-efficacy and the duration per session both showed a significant positive correlation with treatment response of anxiety and depressive symptoms. Self-efficacy, as the core belief in one’s ability to cope, promotes emotional relief through dual pathways. First, patients with high self-efficacy are more inclined to adopt problem-focused coping strategies. When confronted with potential postoperative issues—such as localised swelling or delayed results—they actively seek solutions, a tendency that correlates with reduced accumulation of negative emotions [26]. Second, the manifestation of aesthetic outcomes following treatment further reinforce patients’ self-identity. Postoperative psychological counselling provides patients with a secure channel for emotional expression, helping them release stress arising from unmet aesthetic expectations or concerns about treatment outcomes. Concurrently, healthcare professionals can guide patients to correct cognitive biases—such as excessive focus on minor imperfections or neglect of overall improvement—while imparting emotion regulation techniques. This process intrinsically relates to enhancing patients’ self-management capabilities [27]. Moreover, a potential interaction exists between self-efficacy and psychological counselling. Positive feedback and success stories shared by healthcare professionals during psychological counselling can directly enhance patients’ self-efficacy. Conversely, patients with high self-efficacy are more likely to engage actively with counselling, potentially amplifying intervention outcomes.

Multivariate logistic regression analysis further clarified that the frequency of counselling sessions, duration per session, and preoperative PSQI, MoCA, and GSES scores as constitute factors influencing the treatment response of anxiety and depression. These findings reveal the combined impact of preoperative status and postoperative intervention on emotional outcomes, with the aforementioned variables demonstrating robust predictive efficacy. These findings provide reliable evidence to inform clinical risk stratification and intervention decision-making. Regarding the relative strength of each factor, postoperative psychological counselling emerged as the most prominent modifiable variable for improving emotional outcomes [28]. In contrast, the odds ratio for preoperative PSQI scores was less than 1, indicating that higher scores were corresponded to greater difficulty achieving symptomatic relief. This underscores the need to counteract baseline risk through targeted strategies, such as increasing the frequency of counselling sessions and extending duration per session. Cognitive function provides the foundation for patients to comprehend treatment and engage with interventions, while self-efficacy motivates patients to actively practise emotional regulation techniques and address challenges during recovery [29, 30]. In clinical practice, high-risk individuals can be identified through preoperative questionnaire assessments. For patients with elevated PSQI scores and reduced MoCA or GSES scores, targeted intervention plans can be developed—such as increasing the frequency of psychological counselling to three or more sessions and extending the duration per session to approximately 45 minutes. Such precise, risk-adapted intervention can enhance the effectiveness of emotional therapy.

This study employed a dual design in both outcome definition and analytical strategy. The primary outcome was defined as a composite binary variable based on a ≥50% reduction in both HAMA and HAMD scores, aligning with efficacy assessment criteria widely adopted in psychiatric drug clinical trials; In correlation analyses, HAMA and HAMD reduction rates were incorporated as continuous variables, facilitating a more comprehensive exploration of dose-response relationships between potential influencing factors and the degree of improvement in anxiety and depressive symptoms. These findings demonstrated good consistency between the two analytical approaches. For instance, preoperative PSQI, MoCA, and GSES scores, along with the number and duration per session, showed significant associations with treatment response in both analyses. This indicates that these factors influence symptom improvement not only in terms of achieving clinical remission thresholds but also across the continuous spectrum of symptom reduction. The complementary nature of these analytical approaches, together with the consistency of their findings, further enhances the robustness of the study’s conclusions.

This study has certain limitations. First, as a single-centre retrospective study, all subjects were recruited from a single maternal and child health hospital. The sample predominantly comprised women seeking treatment for cosmetic indications, resulting in a relatively homogeneous distribution in terms of gender and clinical setting. Consequently, extrapolating these findings to male patients, to individuals receiving botulinum toxin for non-cosmetic indications, or to other medical contexts should be undertaken with caution. The retrospective design carries inherent risks of information bias (such as regarding the completeness of medical records) and incomplete control for confounding factors. Second, the follow-up period was limited to eight weeks, which is insufficient to reflect long-term therapeutic responses. The study did not examine the potential interaction between treatment response and satisfaction with cosmetic outcomes. Third, several potential confounding factors—such as social support and personality traits—were not included in the analysis. Fourth, the regression model employed did not specifically adjust for preoperative baseline HAMA and HAMD scores, which may have led to residual confounding effects and could limit the accuracy of the results. Future research should consider multicentre prospective cohort studies that systematically document variables including social support and personality traits, refine psychological counselling protocols, and compare the efficacy of different intervention models. Follow-up periods should be extended to six months or longer, and sample sizes should be increased to enable the construction of more comprehensive predictive models using multicentre. This would provide more robust evidence to inform clinical guideline development.

In summary, this study established correlations between preoperative cognitive function, self-efficacy, and duration per session and treatment response of anxiety and depressive symptoms following botulinum toxin injection. Injection site, frequency and duration per session, and preoperative PSQI, MoCA, and GSES scores were identified as independent factors influencing treatment response. In clinical practice, high-risk individuals can be identified through preoperative screening assessments. Postoperative standardised psychological counselling should be implemented, while reinforcing the protective effects of cognitive function and self-efficacy, to effectively improving patients’ emotional outcomes.

## Conclusions

Preoperative cognitive function, self-efficacy, and the duration per session was correlated with the treatment response of anxiety and depressive symptoms following botulinum toxin injection. Injection site, number of counselling sessions, duration per session, and preoperative PSQI, MoCA, and GSES scores were found as independent factors influencing treatment response. Furthermore, these indicators demonstrated good predictive efficacy when combined in a multivariate model. In clinical practice, emphasis should be placed on managing the mental health of patient receiving botulinum toxin injection. Individualised intervention plans, informed by preoperative assessment, may help optimise emotional outcomes in this populations.

## Availability of Data and Materials

All experimental data included in this study can be obtained by contacting the corresponding author if needed.
